# Payload of T-DM1 binds to cell surface cytoskeleton-associated protein 5 to mediate cytotoxicity of hepatocytes

**DOI:** 10.18632/oncotarget.26461

**Published:** 2018-12-14

**Authors:** Yukinori Endo, Kazuyo Takeda, Nishant Mohan, Yi Shen, Jiangsong Jiang, David Rotstein, Wen Jin Wu

**Affiliations:** ^1^ Division of Biotechnology Review and Research I, Office of Biotechnology Products, Office of Pharmaceutical Quality, Center for Drug Evaluation and Research, U.S. Food and Drug Administration (FDA), Silver Spring, MD, USA; ^2^ Microscopy and Imaging Core Facility, Center for Biologics Evaluation and Research, U.S. Food and Drug Administration (FDA), Silver Spring, MD, USA; ^3^ Division of Compliance, Office of Surveillance and Compliance, Center for Veterinary Medicine, U.S. Food and Drug Administration (FDA), Derwood, MD, USA

**Keywords:** cytoskeleton-associated protein 5 (CKAP5), antibody-drug conjugate (ADC), ado-trastuzumab emtansine (T-DM1), HER2, hepatotoxicity

## Abstract

Off-target toxicity is a major cause of dose-limiting toxicity for antibody-drug conjugates (ADCs), mechanisms of which remain poorly understood. Here, we demonstrate that cytoskeleton-associated protein 5 (CKAP5) serves as a cell surface target for T-DM1 and that binding of T-DM1 to CKAP5 is mediated by payload (DM1). This study introduces a novel molecular mechanism of ADC payload-mediated interaction with cell surface molecules to induce cytotoxicity. Upon binding to CKAP5, T-DM1 causes cell membrane damage and leads to calcium influx into the cells, resulting in disorganized microtubule network and apoptosis. While binding of T-DM1 with HER2 is critical for killing HER2-positive tumor cells, our data suggest that cytotoxicity induced by T-DM1 interaction with CKAP5 may preferentially damage normal cells/tissues where HER2 expression is low or missing to cause off-target toxicity. This study provides molecular basis of ADC-induced off-target cytotoxicity and opens a new avenue for developing next generation of ADCs.

## INTRODUCTION

Antibody-drug conjugate (ADC) is an emerging new class of therapeutic drug for treatments of a variety of cancers [1, 2]. Typically, an ADC consists of three components: monoclonal antibody (mAb) directed against an antigen overexpressed on the cancer cell surface, a cytotoxic payload, and a linker. Upon binding to the cell surface antigen overexpressed on the tumor cell surface via antibody component, ADC is believed to be internalized followed by the release of payload that targets intracellular molecules to mediate cytotoxicity [3, 4]. Ado-trastuzumab emtansine (T-DM1) is an ADC consisting of trastuzumab, a maytansine-derived toxin (DM1), and a nonreducible thioether linker (4-[N-maleimidomethyl]-cyclohexane-1-carbonyl [MCC]). T-DM1 has been approved for treatment of trastuzumab-resistant diseases [5]. Maytansine was originally isolated from an Ethiopian plant, *Maytenus serrata* [6] and has demonstrated antimitotic effects by inhibiting microtubule polymerization [7–9]. Until the emergence of T-DM1, the clinical usage of maytansine had been limited due to the severe toxicity and lack of tumor specificity [10].

ADCs present unique challenges to standard toxicology studies since they consist of both small and large molecule components. This hybrid nature of ADC molecules gives rise to a toxicity profile that is different from that of each individual component. In addition to the impact of conjugation on the pharmacokinetic (PK) profile of payload, which can greatly extend the half-life of a payload, it is also believed that the biodistribution of small drugs such as DM1 is affected by conjugation [11, 12]. In particular, while biodistribution of small molecule payloads generally depends on chemical properties of the molecule, ADCs likely limit the distribution of payloads to where the antibodies are distributed, such as plasma space and antigen-expressing cells/tissues [13, 14].

Hepatotoxicity is the major dose-limiting toxicities observed for T-DM1 during clinical studies [15–18]. ADC instability and antigen-independent uptake by cells are proposed as two major mechanisms of off-target toxicity [18]. The ADC instability refers to premature release of the payload in the circulation resulting in increased systemic exposure to free payloads. However, this mechanism may not apply for T-DM1, since the linker used for T-DM1 is stable in the circulation. The second mechanism is antigen-independent uptake by normal cells. For example, ADCs may be taken up by normal cells through mannose receptors, FcRn, and FcγR receptors expressed on the cell surface [19, 20]. However, these proposals are based on the knowledge obtained from monoclonal antibodies and lack molecular basis that is specific for ADCs.

The mechanisms of T-DM1-induced thrombocytopenia remain controversial. Using a mouse model, Thon et al. reported that T-DM1-induced thrombocytopenia involves HER2- and FcγRIIa-independent pathways, since megakaryocytes/platelets do not express the HER2 and mouse cells do not express the FcγRIIa receptors for human IgGs [21]. Uppal et al. then showed that human megakaryocyte differentiation was inhibited by T-DM1 in HER2-independent, and FcγRIIa-dependent manner [22]. However, Fcγ receptor blocking experiments did not prevent T-DM1 uptake by megakaryocytes [20, 18]. Nevertheless, these studies indicate that there are other non-HER2 and non-FcγR-mediated mechanisms involved in T-DM1-induced toxicity.

Microtubules are critical components of cytoskeleton and widely exploited as major therapeutic targets because of their significant roles in cell migration, trafficking and proliferation [23]. Microtubules consist of heterodimers of α-tubulin and β-tubulin. Because of their integral role in various cellular processes, many microtubule-associated proteins have been identified and characterized [24]. Cytoskeleton-associated protein 5 (CKAP5, also known as ch-TOG or XMAP215) is a member of XMAP215/Dis1 family, which plays a critical role in the regulation of microtubule polymerization. It was reported that CKAP5 directly binds to tubulin via its tumor-overexpressed gene (TOG) domains [25, 26]. It was recently shown that CKAP4 functions as a receptor for the DKK1 to promote cancer cell proliferation [27]. However, it has not been reported that CKAP5 is expressed on the cell surface and serves as T-DM1 target to mediate cytotoxicity to hepatocytes.

## RESULTS

### T-DM1 binds to CKAP5 via its payload, DM1, independent of tubulin

**Figure 1:**
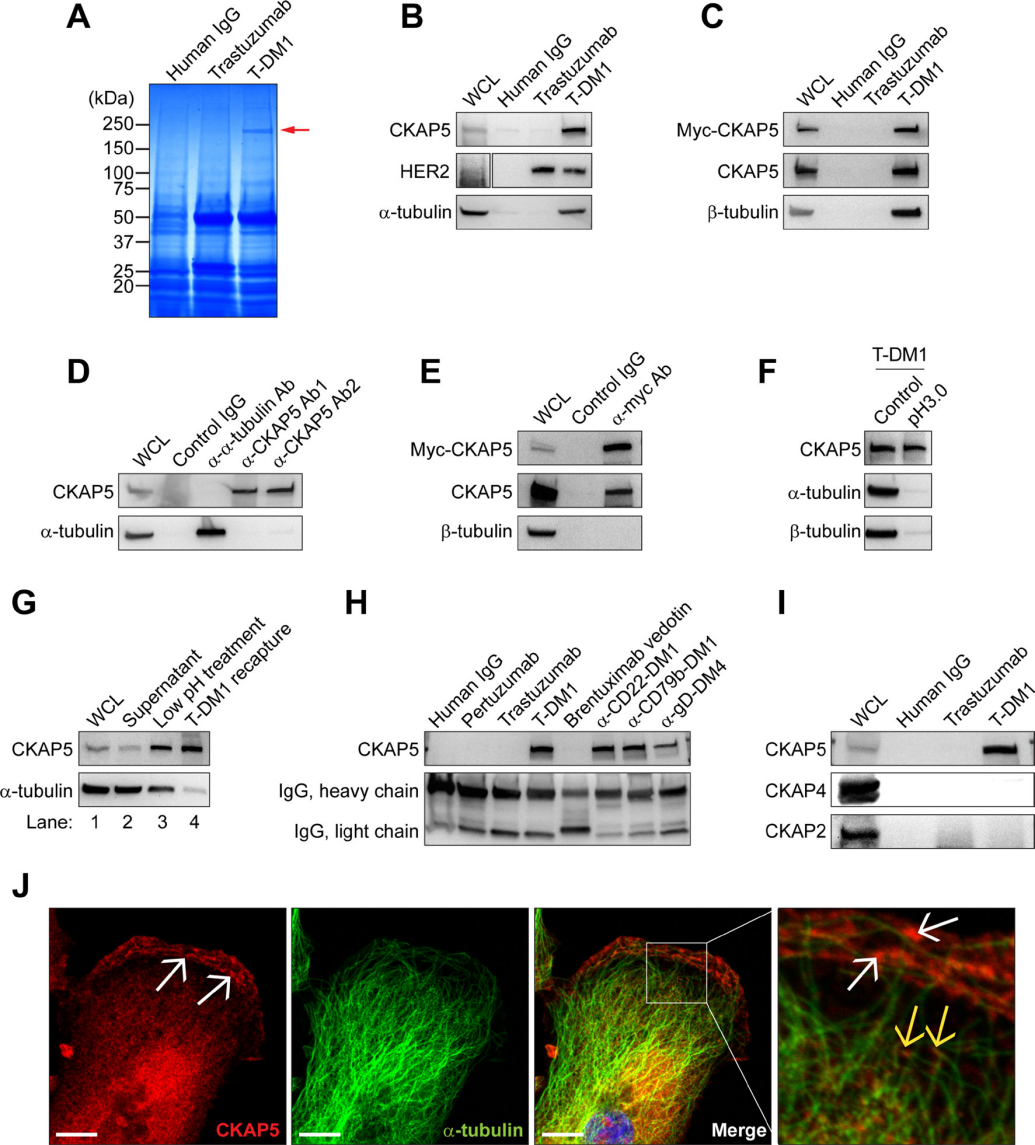
Identification and characterization of CKAP5 as a novel T-DM1-binding protein expressed on the plasma membrane (**A**) Mass spectrometry analysis: A protein band at molecular weight 230 kDa (red arrow) is specifically precipitated by T-DM1, but not by human IgG and trastuzumab. This protein band was further analyzed using mass spectrometry and identified as CKAP5. (**B** and **C**) Immunoprecipitation of CKAP5 by T-DM1 in THLE2 cells: Endogenous (**B**) or exogenously overexpressed myc-tagged (**C**) CKAP5 molecules are immunoprecipitated by T-DM1, not by trastuzumab and control human IgG. The experimental procedures are essentially the same as described in the Materials and Methods for Figure 1A, except that T-DM1 precipitates were subjected to Western blot analysis to probe endogenous or exogenous CKAP5, HER2, α-tubulin or β-tubulin. (**D**) CKAP5 and α-tubulin were unable to co-immunoprecipitate each other. (**E**) Myc-tagged CKAP5 does not co-immunoprecipitate with β-tubulin. WCL generated from myc-tagged CKAP5-overexpressed CHO-K1 cells were subjected to immunoprecipitation using anti-myc antibody. Upper panel: anti-myc blot; middle panel: anti-CKAP5 blot to confirm the immunoprecipitated myc-CKAP5 by anti-myc antibody. (**F**) Low pH 3.0 treatment of WCL abolishes binding of T-DM1 to α-tubulin and β-tubulin, but not to CKAP5 (see Materials and Methods for details). (**G**) CKAP5 interacts with T-DM1 on cell surface independent of α-tubulin (see Materials and Methods for details). (**H**) Maytansine-based ADCs specifically interact with CKAP5 in CHO-K1 cells. After incubation of cells with 100 μg/ml human IgG, pertuzumab, trastuzumab, T-DM1, brentuximab vedotin, anti-CD22-DM1, anti-CD79b-DM1, or anti-gD-DM4 in HBSS at RT for 1 hour, the WCLs were subjected to precipitation using Protein A beads. Western blot for heavy and light chains shows the relative amount of mAbs and ADCs used for the experiments. (**I**) CKAP2 and CKAP4 were not precipitated by T-DM1 in CHO-K1 cells. After incubation with 100 μg/ml human IgG, trastuzumab and T-DM1 in HBSS at RT for 1 hour, the WCLs were subjected to T-DM1 precipitation. (**J**) CKAP5 is found on plasma membrane of THLE2 cells (white arrows) where it does not co-localize with microtubules (yellow arrows). Scale bar, 10 μm.

We previously reported that ADC with DM1 as the payload exhibited HER2-independent and DM1-mediated killing of hepatocytes [28]. To search for novel target molecules that mediate T-DM1-induced off-target cytotoxicity of hepatocytes, T-DM1 (250 μg/ml) was used as a bait and incubated with either human (THLE2) or mouse (AML12) hepatocytes to allow T-DM1 to associate with cell surface molecules. This screen revealed a protein band with relative molecular mass of 230 kDa that specifically binds to T-DM1, but not to trastuzumab or control human IgG (Figure 1A). This 230 kDa protein band was identified by mass spectrometry as CKAP5. Western blot confirmed that CKAP5 was specifically immunoprecipitated (IP) by T-DM1, not by trastuzumab or control IgG (Figure 1B). As expected, both T-DM1 and trastuzumab bound to HER2 (Figure 1B). Figure 1C provided additional evidence that T-DM1 also bound to myc-tagged CKAP5 exogenously expressed in CHO cells. Since DM1 is an established microtubule binding agent [7–9], it was not surprising that both α-tubulin and β-tubulin were co-IPed by T-DM1 (Figure 1B and 1C, respectively).

Using the size-exclusion chromatography, it was shown that CKAP5 forms a complex with free tubulin [26]. Therefore, it is possible that T-DM1 binds to CKAP5 via tubulin. However, α-tubulin failed to co-IP with CKAP5, and little or no α-tubulin was co-IPed with CKAP5 by two different anti-CKAP5 antibodies directed against different epitopes of CKAP5 (Figure 1D). These data were further confirmed by the results showing that the exogenous myc-tagged CKAP5 was also unable to co-IP β-tubulin with CKAP5 by anti-myc antibody (Figure 1E). Figure 1F confirmed that the binding of T-DM1 to CKAP5 is independent of tubulin. Specifically, the whole-cell lysate (WCL) was first treated with low pH (pH 3.0) buffer for 1 to 2 minutes. After neutralized to pH 7.0, the WCL was incubated with T-DM1, and the immunoprecipitates were analyzed by Western blot. While CKAP5 still bound to T-DM1, both α- and β-tubulin were no longer found in T-DM1 immunoprecipitates after low pH treatment. This indicates that low pH treatment of WCL interrupts the interaction between tubulin and T-DM1. Based on this finding we developed another method to address whether the binding of T-DM1 to CKAP5 is independent of tubulin (see Method for details). As shown in Figure 1G, little α-tubulin was found in T-DM1/CKAP5 complex (first lane from the right). Toward this end, we conclude that T-DM1 binds to CKAP5 independent of tubulin.

We next tested whether other maytansinoid-based ADCs bind to CKAP5. As shown in Figure 1H, all maytansinoid-based ADCs, including T-DM1, α-CD22-DM1, α-CD79b-DM1, and α-gD-DM4, bound to CKAP5, whereas brentuximab vedotin, an auristatin (a potent antimiotic agent)-based ADC, trastuzumab, pertuzumab, and control IgG failed to bind CKAP5. Figure 1I showed that both CKAP4, a type II transmembrane protein [29], and CKAP2 were unable to bind to T-DM1.

CKAP5 acts as a microtubule polymerase at the plus ends to accelerate microtubule assembly [25, 26, 30]. Consistent with the previous reports, we found that in addition to punctated cytosolic staining, CKAP5 located at the microtubule plus ends in THLE2 cells (Figure 1J, yellow arrows). Importantly, Figure 1J revealed that CKAP5 also located at the plasma membrane, mostly concentrated at lamellipodia, where it did not co-localize with microtubules on the plasma membrane (white arrows), which supported data showing in Figure 1A that cell surface CKAP5 was immunoprecipitated by T-DM1. This finding raises a question of whether CKAP5 can serve as a target on the cell surface for T-DM1.

### CKAP5 locates on the cell surface and serves as a target for T-DM1

**Figure 2:**
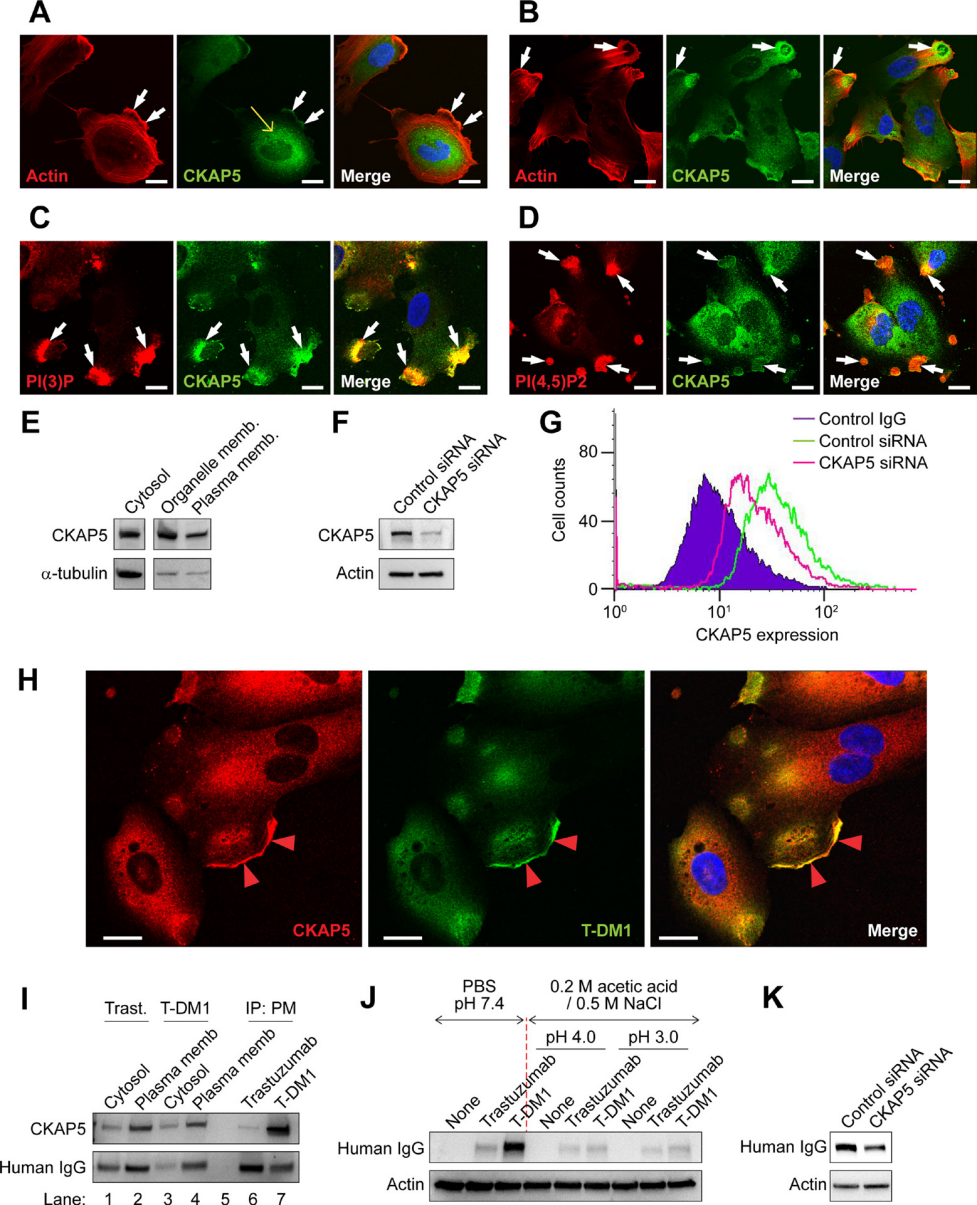
CKAP5 localizes on the cell surface and is targeted by T-DM1 (**A**) Fluorescence immunostaining showing that CKAP5 is co-localized with actin at the cell edges and concentrated at lamellipodia (white arrows) in THLE2 cells. The cells were permeabilized for immunostaining. Scale bar, 20 μm. (**B**, **C** and **D**) Fluorescence immunostaining showing membrane staining of CKAP5 and co-localization of CKAP5 with actin (**B**), PI(3)P (**C**) and PI(4,5)P2 (**D**) on membrane ruffling (white arrows) in THLE2 cells. The cells were not permeabilized for immunostaining. Scale bar, 20 μm. (**E**) Subcellular fractionation of THLE2 cells shows that CKAP5 is found in cytosol, organelle and plasma membrane. (**F**) Knock-down efficiency of CKAP5 expression is evaluated on Western blot. CKAP5 protein expression was about 73% decreased in CKAP5 siRNA-treated THLE2 cells compared with that of control siRNA-treated THLE2 cells. (**G**) Flow cytometric histogram showing that CKAP5 is detected on THLE2 cell surface, and THLE2 cells, in which CKAP5 expression is silenced by siRNA, have lower level of CKAP5 expression than that of control cells. (**H**) Immunostaining of membrane bound T-DM1 and its co-localization with CKAP5 at lamellipodia (red arrowheads) in THLE2 cells. Scale bar, 20 μm. (**I**) T-DM1 binds to CKAP5 from the plasma membrane fraction of THLE2 cells. After incubating 250 μg/ml trastuzumab and T-DM1 with THLE2 cells in HBSS at RT for 1 hour, cell surface proteins were cross-linked by DTSSP and the WCLs were subjected to subcellular fractionation into cytosol and plasma membrane (PM) fraction. PM fraction was solubilized and then subjected to immunoprecipitation. (**J**) T-DM1 specifically binds to the cell surface. The cells were first incubated with 100 μg/ml trastuzumab or T-DM1 in the culture media at 37°C for 1 hr. The cells were then washed with either PBS or low pH stripping buffer containing 0.2 M acetic acid / 0.5 M NaCl (pH 3.0 or 4.0) for 15 secs. Amount of cell-bound trastuzumab and T-DM1 in WCL was detected by Western blot analysis using anti-human IgG conjugated with HRP. (**K**) Amount of cell-bound T-DM1 is examined in WCL of CHO-K1 cells transfected with either control siRNA or CKAP5 siRNA. The experimental procedures are essentially the same as that described in Figure 2J, except that the cells were transfected with indicated siRNA and were not treated with low pH buffer.

Immunolocalization of CKAP5 showed that while CKAP5 displayed as punctate structures throughout the cytoplasm in THLE2 cells (Figure 2A, yellow arrow) and SKBR3 breast cancer cells (Supplementary Figure 1A, yellow arrow), it was also found at the cell periphery where it co-localized with actin (Supplementary Figure 1A, white arrows) and particularly concentrated at the lamellipodia of THLE2 cells (Figure 2A, white arrows). To address whether CKAP5 locates on the cell surface outside of the plasma membrane, cells used for the experiments were not permeabilized for the purpose of immunostaining of cell surface CKAP5 (Figure 2B, 2C and 2D, and Supplementary Figure 1B). CKAP5 was detected on the cell surface of THLE2 cells and concentrated at the lamellipodia (Figure 2B, white arrows) and the membrane ruffles (Supplementary Figure 1B, white arrows). CKAP5 was also found to co-localize with phosphatidylinositol (4,5)-bisphosphate (PIP2) and phosphatidylinositol 3-phosphate (PI3P) in THLE2 cells, both of which are essential components of plasma membrane [31] (Figure 2C and 2D).

Subcellular fractionation analysis supported that CKAP5 localized in the plasma membrane (Figure 2E and Supplementary Figure 1C). Based on the subcellular fractionation experiment (*n* = 4), the amount of CKAP5 protein detected in plasma membrane and cytosolic were 31% ± 7.5% and 68% ± 12.4%, respectively. Importantly, flow cytometry analysis without cell permeabilization detected strong positive signals of CKAP5 exposed on the cell surface of THLE2 and CHO cells, and these positive signals were decreased in CKAP5 knock-downed THLE2 and CHO-K1 cells compared with that of the control siRNA cells (Figure 2F and 2G, and Supplementary Figure 1D and 1E). Moreover, tubulin was not detected on the cell surface of THLE2 and CHO cells (Supplementary Figure 1F and 1G). Supplementary Figure 1H showed that CKAP5 expression determined by Western blot analysis in THLE2 cells was higher than that in human cardiomyocytes (HCM), and cell surface expression of CKAP5 determined by flow cytometry analysis of THLE2 and HCM cells was consistent with data shown in Supplementary Figure 1H (Supplementary Figure 1I). These data may implicate that more T-DM1 molecules likely accumulate in liver than heart tissues through binding to CKAP5. Taken together, our data indicate that CKAP5 is a cell surface protein.

We next investigated whether T-DM1 binds to CKAP5 on the cell surface. THLE2 cells were treated with T-DM1 for 1 hour and then were subjected to immunostaining without permeabilization. As shown in Figure 2H, T-DM1 was found on the cell surface and accumulated at the lamellipodia where it co-localized with CKAP5 (arrowheads). We next addressed the interactions between CKAP5 and T-DM1 on the cell surface using a biochemical approach. THLE2 or CHO-K1 cells were incubated with either trastuzumab or T-DM1 at room temperature for 1 hour and then the cell surface proteins were cross-linked with DTSSP (see Materials and Methods). The levels of CKAP5, trastuzumab, T-DM1 in cytosol or plasma membrane fractions were examined (Figure 2I and Supplementary Figure 1J, lanes 1 to 4). Trastuzumab and T-DM1 in the different fractions were detected using anti-human IgG secondary antibody. The plasma membrane fraction was solubilized using NP40 containing buffer and then subjected to Protein A affinity precipitation. As shown in Figure 2I (THLE2) and Supplementary Figure 1J (CHO) (lanes 6 and 7), CKAP5 was detected in T-DM1, not trastuzumab, Protein A precipitates, indicating that CKAP5 bound to and was cross-linked with T-DM1 at the cell surface.

To further confirm if T-DM1 associates with CKAP5 on the cell surface, after incubating cells with trastuzumab or T-DM1 for 1 hr, the cell surface bound proteins were stripped with the acidic stripping buffer and analyzed by Western blot. We found that amount of T-DM1 was diminished from the cells (Figure 2J), indicating that binding of T-DM1 to the cells occurred on the cell surface. When the expression of CKAP5 was silenced by siRNA (Note: The knock-down efficiency of CKAP5 was shown in Supplementary Figure 1D), binding of T-DM1 to the cells, which was detected by anti-human IgG antibody, was decreased in CKAP5 knock-downed cells compared with that of control cells (Figure 2K), suggesting that T-DM1 binds to cell surface in a CKAP5-dependent manner. Furthermore, Supplementary Figure 1K shows that the amount of T-DM1 binding to the cells was inhibited by pre-incubation of anti-CKAP5 antibody (α-CKAP5) that was used for flow cytometry (Figure 2G), but not by the anti-CKAP5 antibody (α-CKAP5 Ab1) that was not capable of detecting cell surface expression of CKAP5 (data not shown). These data strongly support that CKAP5 localized on the cell surface is responsible for T-DM1 binding to the cells.

### T-DM1 inhibits microtubule assembly accompanied by actin cytoskeleton rearrangement

**Figure 3:**
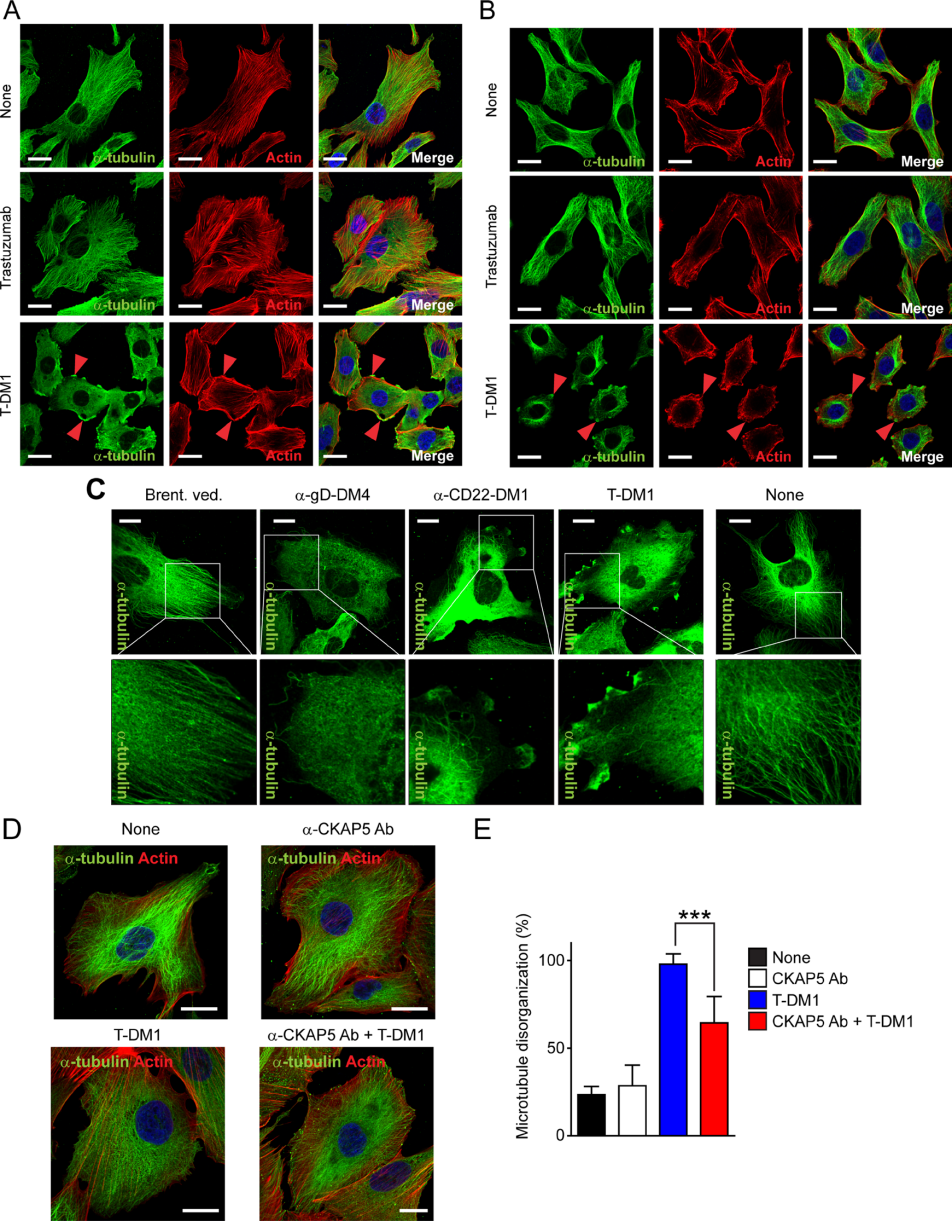
T-DM1 inhibits microtubule assembly and anti-CKAP5 antibody prevents T-DM1 from inducing microtubule disassembly in THLE2 cells (**A** and **B**) T-DM1 induces microtubule disassembly accompanied by actin cytoskeleton disorganization in THLE2 cells (**A**) and CHO cells (**B**). the cells were incubated with 100 μg/ml trastuzumab and T-DM1 for 3 hours and then were fixed for fluorescence immunostaining of α-tubulin (green) and actin (rhodamine phalloidin, red). Nucleus was stained with DAPI (blue). Scale bar, 20 μm. (**C**) Maytansinoid-based ADCs induces microtubule disassembly in THLE2 cells. Cells were incubated with indicated ADCs at 100 μg/ml for 1 hr. Non-maytansinoid-based ADC, brentuximab vedotin, was used as a control. Cells were fixed and permeabilized for fluorescent immunostaining for CKAP5, α-tubulin and nucleus. Scale bar, 20 μm. (**D**) Anti-CKAP5 antibody prevents T-DM1 from inducing microtubule disassembly in THLE2 cells. THLE2 cells were seeded on fibronectin-coated cover glass were pre-incubated with 25 μg/ml anti-CKAP5 antibody (Cat# HPA040375, Sigma-Aldrich) at 37°C for 1 hour and were then treated with 25 μg/ml T-DM1 for 1 hour. Cells then were subjected to immunostaining. Scale bar, 20 μm. (**E**) Quantification of THLE2 cells with the disrupted microtubule networks caused by T-DM1. Cell numbers showing disrupted microtubule networks were quantified per total cell numbers counted in randomly selected fields (*n* = 5 to 8, total 71 to 172 cells).

We next investigated whether binding of T-DM1 to CKAP5 on the cell surface impacted on biophysiological cell functions. After 3 hrs incubation of THLE2 (Figure 3A) or CHO-K1 (Figure 3B) cells with T-DM1, the microtubule structures were completely diminished as compared with that in control and trastuzumab-treated cells. Moreover, microtubule disassembly was accompanied by actin cytoskeleton redistribution and accumulation at the cell edges, especially at lamellipodia where CKAP5 is often found to co-localize with T-DM1 (Figure 2H) and the loss of stress fibers (Figure 3A and 3B, arrowheads).

### Maytansinoid-based ADC interrupts microtubule polymerization and anti-CKAP5 antibody prevents T-DM1 from inhibiting microtubule assembly

We then asked a question of whether other maytansinoid-based ADCs, which do not target a specific antigen expressed on the cell surface and are unlikely to be internalized via antibody/antigen interactions on the cell surface, are able to inhibit microtubule assembly in hepatocytes. THLE2 cells were treated with anti-CD22-DM1 and anti-gD-DM4 (an anti-herpes simplex virus (HSV) glycoprotein ADC) for 1 hr. Both CD22 and HSV are not expressed in human hepatocytes according to Human Protein Atlas (http://www.proteinatlas.org/). As shown in Figure 3C, both anti-CD22-DM1 and anti-gD-DM4 inhibit microtubule assembly in THLE2 cells. However, this was not the case when THLE2 cells were treated with brentuximab vedotin, an ADC that consists of anti-CD30 antibody and MMAE and is unable to bind to CKAP5 (Figure 3C). These data indicate that the ability of DM1 or DM4-based conjugates to inhibit microtubules assembly is DM1/DM4 dependent, rather than non-specific uptake of ADCs into the cells. To further confirm that binding of T-DM1 to the cell surface CKAP5 is necessary for T-DM1 to induce microtubule disassembly, THLE2 cells were pre-incubated with anti-CKAP5 antibody for 1 hr. The cells then were treated with T-DM1 for 1 hr. As shown in Figure 3D, no morphological changes were observed when cells were treated with either control or anti-CKAP5 antibody for 1 hr. As expected, cells treated with T-DM1 displayed microtubule disassembly. However, T-DM1-induced microtubule disassembly were significantly reduced when cells were pre-incubated with anti-CKAP5 antibody (Figure 3E). These data provide strong evidence that the specific interaction between T-DM1 and CKAP5 plays an important role in mediating T-DM1-induced microtubule disassembly.

### Silencing CKAP5 mimics the effects of T-DM1 on microtubule networks, resulting in nucleus fragmentation and cell growth inhibition

**Figure 4:**
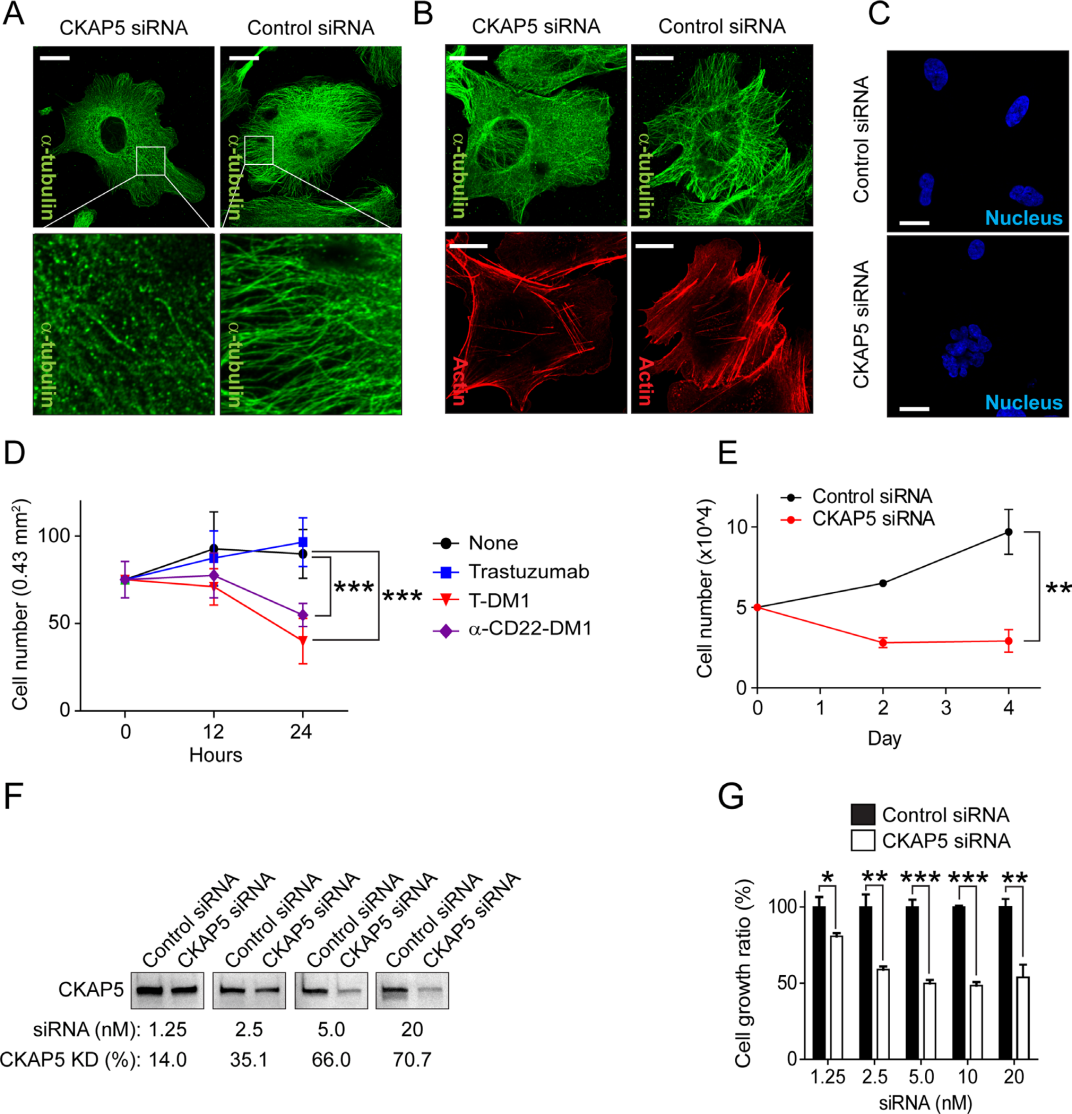
Silencing CKAP5 interrupts microtubule networks, resulting in nucleus fragmentation and THLE2 cell growth inhibition (**A**) Fluorescent immunostaining showing that microtubule polymerization is disrupted in CKAP5 knock-downed THLE2 cells. Scale bar, 20 μm. (**B**) Fluorescent immunostaining showing that microtubule polymerization and actin cytoskeleton are interrupted in CKAP5 knock-downed CHO-K1 cells after 72 hours post siRNA transfection. Scale bar, 20 μm. (**C**) Fluorescent immunostaining showing that nucleus is fragmented in CKAP5 knock-downed CHO-K1 cells after 72 hrs post siRNA transfection. Scale bar, 20 μm. (**D**) THLE2 cells were incubated with 100 μg/m trastuzumab, brentuximab vedotin, T-DM1, or α-CD22-DM1 at 37°C for 0, 12, 24 hours. Then, cells were fixed for fluorescence immunostaining of actin (rhodamine phalloidin). Nucleus was stained with DAPI. Total survived cell number was counted in randomly selected area (4.3 × 10^5^ μm^2^, each *n* = 7) captured by a Zeiss LSM880 confocal microscope. (**E**) Growth profiles of THLE2 cells. The cells were transfected with either control siRNA or CKAP5 siRNA. 48 hrs post siRNA transfection, 5 × 10^4^ THLE2 cells were seeded in 12-well plates and incubated in the cell culture media containing 10% serum. On the indicated days, cells were trypsinized and counted. (**F**) Western blot showing CKAP5 knock-down efficiencies with various concentrations of siRNA. 48 hrs post siRNA transfection, CKAP5 expression in THLE2 cells was examined using Western blot analysis. (**G**) CKAP5 silencing inhibits THLE2 cell growth that is correlated with the levels of CKAP5 expression in THLE2 cells. THLE2 cells were transfected with either control siRNA or CKAP5 siRNA and incubated in the culture media containing 10% FBS. 48 hrs post siRNA transfection, 5 × 10^4^ cells/well were seeded in a 12-well plate. Then, the cells were counted on Day 2.

Although cell surface CKAP5 serves as a target of T-DM1 to mediate microtubule disruption in hepatocytes, as described previous sections, CKAP5 are also localized along with microtubules inside of the cells and plays an important role in the regulation of microtubule polymerization. Thus, the roles of CKAP5 on the regulation of microtubule and actin cytoskeleton rearrangement were further assessed by CKAP5 knock-down. Silencing CKAP5 interrupted microtubule and actin cytoskeleton networks, resulting in microtubule punctuation, actin cytoskeleton rearrangement to the cell periphery, and nucleus fragmentation (Figure 4A, 4B and 4C). Figure 4D showed that DM1-conjugated ADCs inhibited THLE2 cell growth. The same was true when CKAP5 expressing was silenced in THLE2 cells (Figure 4E, 4F and 4G).

### Binding of T-DM1 to CKAP5 on the cell surface is independent of HER2

**Figure 5:**
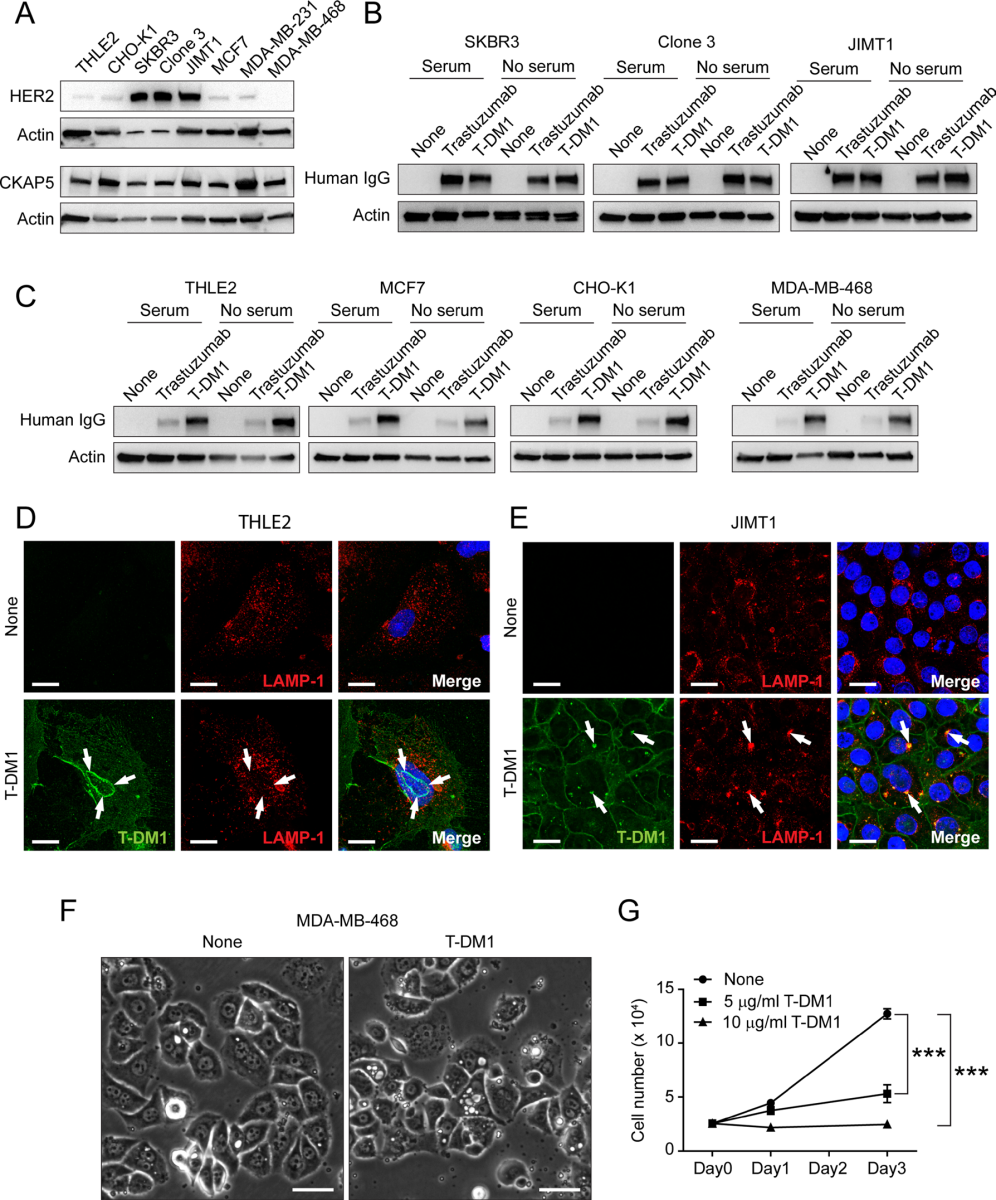
Binding of T-DM1 to cell surface CKAP5 is independent of HER2 (**A**) The levels of endogenous HER2 and CKAP5 in the WCL of the indicated cell lines analyzed by Western blot. (**B** and **C**) Binding of trastuzumab and T-DM1 to the breast cancer cells overexpressing HER2 (**B**) or cells expressing low levels of HER2 (**C**). The indicated cell lines were incubated with 100 μg/ml trastuzumab or T-DM1 in the culture media either with or without serum at 37°C for 1 hr. Amount of trastuzumab and T-DM1 in WCL were determined by Western blot analysis using anti-human IgG conjugated with HRP. (**D**) T-DM1 is not co-localized with LAMP-1 in THLE2 cells. THLE2 cells were incubated with 100 μg/ml trastuzumab or T-DM1 for 1 hour at 37°C, and subsequently were fixed for fluorescent immunostaining of trastuzumab or T-DM1 (green) and LAMP-1 (red). Nucleus was stained with DAPI (blue). Images were taken by a Zeiss LSM880 confocal microscope. Scale bar, 20 μm. (**E**) T-DM1 is co-localities with LAMP-1 in JIMT1 cells. JIMT1 cells were incubated with 100 μg/ml T-DM1 for 1 hour at 37°C, and subsequently were fixed for fluorescent immunostaining of T-DM1 (green) and LAMP-1 (red). Nucleus was stained with DAPI (blue). Images were taken by a Zeiss LSM880 confocal microscope. Scale bar, 20 μm. (**F**) Bright field images showing cytoplasmic vacuolization induced by T-DM1 in MDA-MB-468 cells that do not express HER2. Scale bar, 40 μm. (**G**) Cell growth profiles of MDA-MB-468 cells. Cells were treated with indicated dose. Specifically, 2.5 × 10^4^ cells/well were seeded in 12-well plates and incubated in the cell culture media containing either 5 μg/ml or 10 μg/ml of T-DM1. On the indicated days, the cells were trypsinized and counted.

Figure 5A showed the levels of HER2 and CKAP5 expression among different cell lines. Based on different expression levels of HER2 and CKAP5, we developed an assay to address whether binding of T-DM1 to the cell surface CKAP5 is dependent on HER2 (Refer to Method for the detailed description of experimental design). We found that similar amounts of trastuzumab and T-DM1 associated with the cells (SKBR3, Clone3 and JIMT1, all of which are HER2-positive breast cancer cell line.) where HER2 is overexpressed, suggesting that trastuzumab and T-DM1 predominantly associate with HER2 (Figure 5B). In contrast, the amount of trastuzumab binding to the cells was much lower than that of T-DM1 in the cells with low levels of HER2 expression, suggesting that binding of T-DM1 to the cell surface CKAP5 contributes this difference (Figure 5C). These data confirm that T-DM1 associates with CKAP5 on the cell surface independent of HER2. Figure 5D and 5E showed that THLE2 cells did not sufficiently internalize T-DM1 as compared to HER2-positive JIMT1 cells such that T-DM1 largely stayed on the THLE2 cells surface, rather than internalized and colocalized with the lysosomal marker, LAMP-1, as displayed in JIMT1 cells. Figure 5F showed that T-DM1 induced cytoplasmic vacuolization, a typical morphological phenomenon that is often observed in mammalian cells after exposure to low-molecular-weight compounds and accompanied with cell death [32], in MDA-MB-468 cells, which do not express HER2 and was purposely chosen to rule out HER2-mediated effects on the cells. Figure 5G showed HER2-independent and dose-dependent cell growth inhibition of MDA-MB-468 cells induced by T-DM1. These data suggest a mechanism of T-DM1 action to kill cells that is independent of binding to HER2 and is likely mediated by CKAP5.

### T-DM1 damages plasma membrane of THLE2 cells, leading to calcium influx and apoptosis

**Figure 6:**
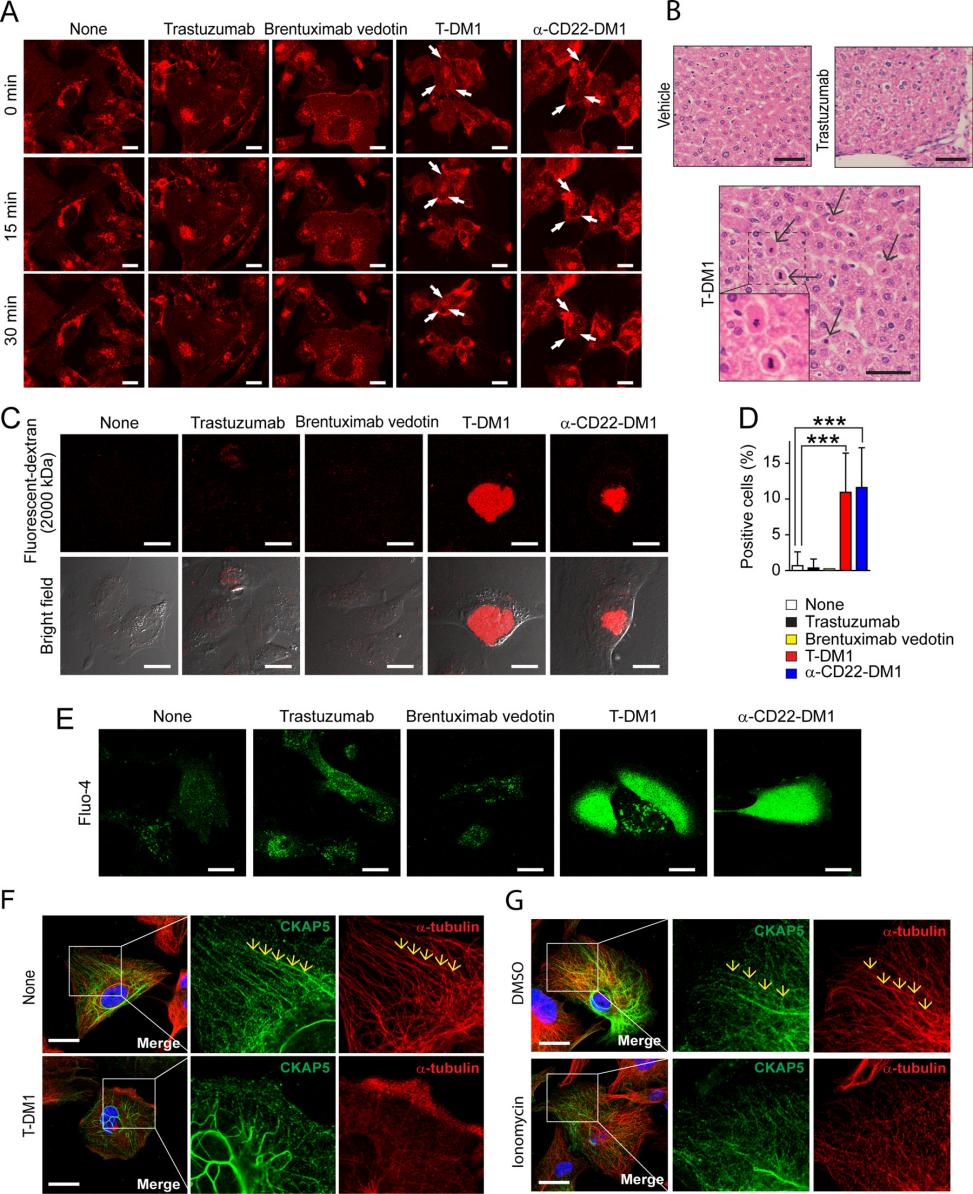
T-DM1 damages plasma membrane, leading to calcium influx and microtubule disorganization and apoptosis (**A**) T-DM1 causes rapid cell shrinkage in size and membrane blebs in THLE2 cells. Human hepatocytes (THLE2) cells were incubated with 100 μg/ml trastuzumab, brentuximab vedotin, T-DM1 or α-CD22-DM1 at 37°C for 45 mins, and then rhodamine-conjugated wheat germ agglutinin (WGA) was added to cell culture for 15 mins. After washing cells with the cell culture media twice, time-lapse recording was performed for 30 mins using a Leica SP8 DMI 6000 (Supplementary Video 1-none, 2-trastuzumab, 3-brentuximab vedotin, 4-T-DM1, and 5-α-CD22-DM1). Images shown were taken at 0, 15, and 30 min time-points. T-DM1 and α-CD22-DM1 caused rapid cell shrinkage and blebs, whereas trastuzumab and brentuximab vedotin didn’t. Scale bar, 20 μm. (**B**) T-DM1 administration induces apoptosis of mouse liver cells. Liver tissue was collected on Day3 from the mice with single dose tail vein injection of vehicle control, 29.4 mg/kg trastuzumab and 3 mg/kg T-DM1. The harvested liver tissues were used for H&E staining. Scale bar, 50 μm. (**C**) T-DM1 induces plasma membrane damage in THLE2 cells. THLE2 cells were incubated with 100 μg/ml trastuzumab, brentuximab vedotin, T-DM1 or α-CD22-DM1 together with 125 μg/ml tetramethylrhodamine-conjugated 2,000,000 Da dextran at 37°C for 1 hour. After washing cells with the culture media twice, cell membrane damage was monitored by observing the fluorescent dextran taken up by cells using a Leica SP8 DMI 6000 confocal microscope. T-DM1 and α-CD22-DM1 caused accumulation of the fluorescent dextran in the treated cells. Scale bar, 20 μm. (**D**) Quantification of 2,000,000 Da dextran-positive cells. The dextran-positive cells were quantified in randomly selected area (number of area from each sample is from 11 to 16, total 190 to 312 cells were counted for each samples) and captured by a Leica SP8 DMI 6000. (**E**) T-DM1 induces calcium influx in THLE2 cells. THLE2 cells were incubated with Fluo-4 for 30 mins, and subsequently were incubated with 100 μg/ml trastuzumab, brentuximab vedotin, T-DM1, or α-CD22-DM1 at 37°C for 1.5 hrs. Calcium influx (green) was observed using a Leica SP8 DMI 6000 confocal microscope. Scale bar, 20 μm. (**F**) T-DM1 induces rapid microtubule disassembly in THLE2 cells. THLE2 cells were incubated with 100 μg/ml T-DM1 for 1 hour and then were fixed with methanol for fluorescent immunostaining for CKAP5, α-tubulin and nucleus (see Materials and Methods). Scale bar, 20 μm. (**G**) Ionomycin-induced calcium influx causes microtubule disassembly in THLE2 cells. The cells were incubated with 5 μM ionomycin at 37°C for 1 hour and then were fixed for fluorescence immunostaining of α-tubulin (red) and CKAP5 (green). Nucleus was stained with DAPI (blue). Images were taken by a Zeiss LSM880 confocal microscope. Scale bar, 20 μm.

We then asked question of how T-DM1-CKAP5 interaction on the cell surface induces cytotoxicity. We performed a live imaging experiment, which included brentuximab vedotin to control possible non-specific uptake of ADC molecules by THLE2 cells. α-CD22-DM1, a DM1-conjugated ADC, was also included in this experiment since THLE2 cells do not express CD22. As shown in Figure 6A and Supplementary Videos 1, 2, 3, 4, and 5, both T-DM1 and α-CD22-DM1 induced cell shrinkage and membrane blebs, typical morphological changes of apoptosis, in THLE2 cells, whereas brentuximab vedotin, trastuzumab, mock treatment failed to change cell morphology of THLE2 cells. These data indicate that the morphological changes of THLE2 cells induced by T-DM1 or α-CD22-DM1 are DM1-dependent. Figure 6B showed that apoptotic cells were only found in liver tissues from mice treated with T-DM1.

Since THLE2 cells do not sufficiently internalize T-DM1, then question is how T-DM1 effectively induced apoptosis of THLE2 cells. It could be that T-DM1 may use a mechanism yet unidentified, which is different from the conventional mechanism of action of ADC. We incubated THLE2 cells with either T-DM1 or α-CD22-DM1 with 2,000,000 Da fluorescent-dextran as the flux tracer to measure whether DM1-conjugated ADC can damage plasma membrane. Since the molecular weight of dextran is 2,000,000 Da, this can rule out the possibility of normal membrane trafficking processes that could non-specifically uptake the flux tracer inside of the cells. Therefore, if we can detect 2,000,000 Da fluorescent-dextran inside of the cells, it indicates that DM1-conjugated ADCs are capable of damaging plasm membrane upon binding to cell surface CKAP5. As shown in Figure 6C and 6D, significant amount of 2,000,000 Da fluorescent-dextran was only found in the hepatocytes treated with either T-DM1 or α-CD22-DM1, not trastuzumab or brentuximab vedotin. Using a calcium indicator Fluo-4, we found that the membrane damage caused by DM1-conjugated ADCs led to a dramatic calcium influx inside of hepatocytes (Figure 6E). Using methanol fixation for fluorescent immunostaining We found that CKAP5 is localized along with microtubules (Figure 6F). Using the same method, we found that T-DM1 treatment disrupted microtubules organization and caused disengagement of CKAP5 from microtubules (Figure 6F, lower panels). Ionomycin is a calcium ionophore that can transport calcium cross plasma membranes and raise the intracellular level of calcium. As shown in Figure 6G, cells treated with ionophore displayed similar morphological changes to that induced by T-DM1, such that microtubule network was disassembled and CKAP5 molecule was disengaged from the microtubules. Taken together, our results indicate that CKAP5 not only serves as a target for T-DM1 on cell surface, but also plays an important role in the regulation of microtubules.

## DISCUSSION

ADCs are generally developed with the purpose of delivering the potent toxic payload specifically to antigen-overexpressed tumor cells to increase the therapeutic index (TI) while considerably reducing damage to normal tissues [19]. However, off-target or antigen independent toxicity is a major cause of dose-limiting toxicity (DLT) for ADC therapeutics [18]. Furthermore, for most ADCs conjugated to microtubule inhibitors, e.g. DM1 and DM4, the maximum tolerated dose (MTD) is between 1.8 to 5.8 mg/kg irrespective of antigen expression [33]. Therefore, improving TI of next generation ADCs will not be accomplished unless we obtain a greater understanding of the underlying mechanisms of off-target toxicity [18].

**Figure 7:**
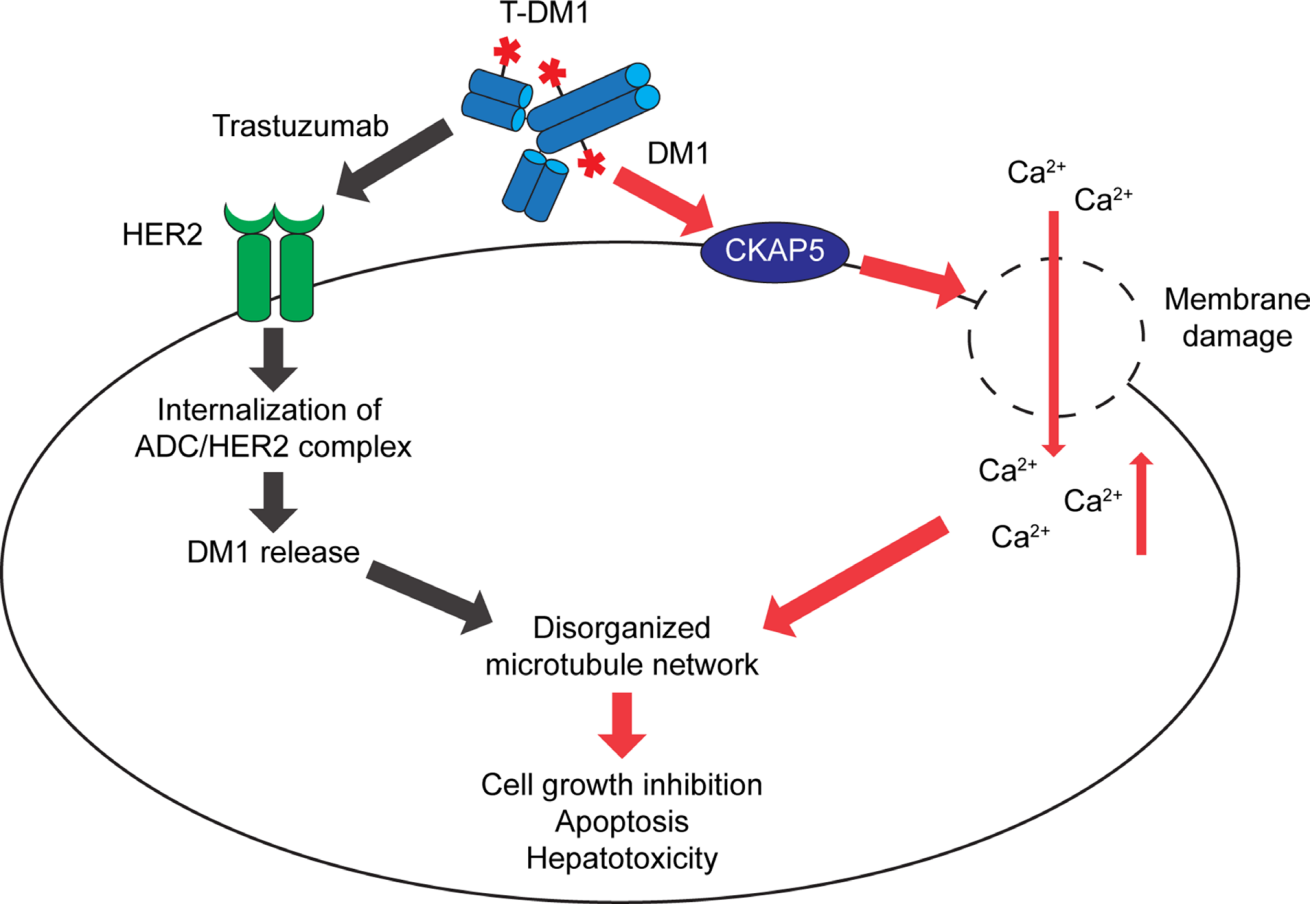
A working model: The payload (DM1)-mediated cytotoxicity Upon specific binding to CKAP5 on cell surface of hepatocytes, T-DM1 begins to damage cell plasma membrane followed by osmolality changes inside of hepatocytes due to calcium influx. Increased calcium concentration in the cytosol causes disorganized microtubule network, resulted in cell growth inhibition, apoptosis and hepatotoxicity. While T-DM1 antibody-mediated interaction with HER2 plays a major role for ADC-induced killing in HER2-positive tumor cells, payload (DM1)-mediated cytotoxicity is particularly important for T-DM1-induced cytotoxicity of normal cells/tissues where HER2 expression is low or missing to induce “off-target” toxicity.

In the HER2-overexpressing breast cancer cells, upon binding to HER2, T-DM1/HER2 complex is quickly internalized following by lysosomal degradation of antibody portion, leading to the intracellular release of payload, lys-MCC-DM1, to target microtubules and mediate apoptosis and cell death [12, 34, 35]. The present study reveals a novel mechanism by which T-DM1-induced cytotoxicity of normal cells. Specifically, we provide strong evidence demonstrating that CKAP5 serves as a cell surface target for T-DM1, and binding of T-DM1 to CKAP5 is via DM1 and independent of tubulin and HER2. A working model for T-DM1-induced cytotoxicity of liver cells, based on a number of lines of experimental evidence, is presented in Figure 7. Upon specific binding to CKAP5 on the cell surface, T-DM1 begins to damage cell plasma membrane, leading to dramatic calcium influx into the liver cells. Increased calcium concentration in the cytosol changes osmolality inside of the hepatocytes and causes disorganized microtubule network, resulting in cell growth inhibition, apoptosis and cell death. While T-DM1 antibody-mediated interaction with HER2 plays a major role for T-DM1-induced killing of HER2-positive tumor cells, T-DM1 payload-mediated cytotoxicity particularly impacts on normal cells and tissues where HER2 expression is low or missing to induce antibody independent toxicity.

Our study reveals a novel mechanisms of ADC-mediated, antigen-independent cytotoxicity of normal cells/tissues. The internalization of ADC (e.g., T-DM1) followed by payload (e.g., DM1) release inside of the cells to target intracellular microtubules may not be necessary steps for T-DM1-induced cytotoxicity in normal cells, such as hepatocytes. The significance of this novel mechanism of ADC payload-mediated cytotoxicity is particularly important for normal cells/tissues where antigen expression is low or missing and is insufficient to induce ADC/antigen complex internalization. This study provides evidence that explains why ADCs can effectively inhibit growth of hepatocytes, which have no or low specific antigens expressed on the cell surface, in a dose-dependent manner [28].

DM1-mediated interaction of T-DM1 with CKAP5 may also change our current understanding of ADC biodistribution, which is believed to be largely determined by the interactions between antibody and antigen-expressing cells/tissues. While CKAP5 is ubiquitously expressed (http://www.proteinatlas.org/ENSG00000175216-CKAP5/tissue), its expression varies in different tissues. Therefore, its expression levels on cells surface in different tissues needs to be taken into the consideration for ADC biodistribution as it may contribute to the organ-specific toxicity. The present study advances our understanding of mechanisms for ADC-induced cytotoxicity and challenges the conventional concept for the mechanisms of ADC action when it targets normal cells to induce unwanted toxicity. The design of next generation of ADCs should take into consideration minimizing payload-mediated, antigen-independent toxicity to the normal cells.

## MATERIALS AND METHODS

### Mouse maintenance

All animal experiments were approved by and conducted in accordance with the regulation of the FDA Institutional Animal Care and Use Committee guidelines. The detail procedures were described in our previous study [28]. Briefly, C57BL/6 mice were randomly assigned into five groups, including vehicle, trastuzumab (29.4 mg/kg), T-DM1 (3 mg/kg), T-DM1 (10 mg/kg), or T-DM1 (30 mg/kg) for the time and dose-dependent study. Mice received a single tail-vein injection of vehicle, trastuzumab, or T-DM1. Livers from mice were harvested for H&E staining [28].

### Tissue culture cells

THLE2 and CHO-K1 cells were cultured in media containing 10% FBS and antibiotics at 37°C and 10% CO_2_. All breast cancer cell lines except JIMT1 were purchased from American Type Culture Collection (ATCC) and cultured in the serum containing media according to the instructions provided by ATCC. JIMT1 was purchased from DSMZ and cultured in DMEM containing 10% FBS according to the instructions provided by the company. Transfections of cells with either siRNA or plasmid DNA were performed according manufacturer's instructions.

### Identification of T-DM1-binding protein using SDS-PAGE and mass spectrometry

THLE2 or AML12 cells (80–90% confluency in 15-cm dishes) were incubated with 250 μg/ml human IgG, trastuzumab or T-DM1 in Hanks’ balanced salt solution (HBSS, Thermo Fisher Scientific) at room temperature for 1 hour. Subsequently, cells were lysed with NP40 lysis buffer on ice for 30 mins, and the whole-cell lysate (WCL) was subjected to immunoprecipitation using Protein A agarose (Sigma-Aldrich) to capture human IgG, trastuzumab and T-DM1. After washing the Protein A agarose beads with NP40 lysis buffer three times, the immunoprecipitates were eluted by 1 x Laemmli sample buffer (BioRad) containing 2-mercaptoethanol (Sigma-Aldrich) at 95°C for 10 mins and then subjected to SDS-PAGE analysis. A protein band at about 230 kDa was specifically immunoprecipitated by T-DM1, but not by control human IgG and trastuzumab. This band was excised from SDS-PAGE gel and further analyzed by mass spectrometry (MS). This 230 kDa was identified as CKAP5 based on MS score (see Supplementary Materials).

### H&E staining

The detailed experimental procedures for H&E staining were described previously [28].

### Assays to determine HER2-independent binding of T-DM1 on the cell surface

5 × 10^5^ cells/well were seeded in 6-well plates (Corning) and incubated in cell culture media containing 10% FBS for 24 hrs. The cells were then incubated overnight with fresh media containing either 0% or 10% FBS. The cells were treated with either 100 μg/ml trastuzumab or T-DM1 at 37°C for 1 hour. The cells then were washed with PBS for at least three times [Note: In some experiments (Figure 2J), cells were treated with a stripping buffer (0.2 M acetic acid / 0.5 M NaCl at pH 3.0 or 4.0) for 15 secs after washing by PBS.], cells were collected using 0.05% trypsin-EDTA (Thermo Fisher Scientific) and lysed in NP40 lysis buffer on ice for 30 mins. The WCL was subjected to Western blot analysis to determine the amounts of trastuzumab and T-DM1 using horseradish peroxidase (HRP) conjugated anti-human IgG secondary antibody (EMD Millipore).

### Immunofluorescent staining

Cells were plated on either non-coated or fibronectin (10 μg/ml at 4°C overnight, Sigma-Aldrich) pre-coated glass coverslips in 12-well plate and cultured overnight. Without washing, cells were fixed in 4% paraformaldehyde (Electron Microscopy Sciences, cat#15710) for 20 mins and permeabilized with either 0.2% TritonX-100 / TBS or 0.5% Saponin (EMD Millipore) / TBS for 10 mins. After blocking with 10% donkey serum (Jackson ImmunoResearch), cells were subjected to fluorescent immunostaining. For immunostaining to detect T-DM1 on the cell surface, cells plated on fibronectin pre-coated cover glass were fixed and washed with PBS without permeabilization. After blocked with 10% donkey serum in TBS at 37°C for 30 mins, the cells were subjected to immunofluorescence staining. ProLong Gold antifade reagent with DAPI (Thermo Fisher Scientific) was used for mounting specimens on glass slides and nucleus staining. Images were captured by an LSM 880 confocal microscope (Carl Zeiss Microscopy).

### CKAP5 plasmid DNA transfection

Full-length human CKAP5 tagged with myc epitope was synthesized by Genewiz and then subcloned into pCAGGS vector [36] by Genewiz. The CKAP5 expression plasmid was transfected into CHO-K1 cells using 4D-Nucleofector (Lonza). 48 hrs post transfection, cells were used for immunoprecipitation.

### Plasma membrane fractionation and protein A agarose bead precipitation (Figure 2I and Supplementary Figure 1J)

Four to five of 15-cm (Corning) dishes of 90–100% confluent of THLE2 or CHO-K1 cells were prepared. After washing with HBSS twice, cells were incubated with 250 μg/ml either trastuzumab or T-DM1 in HBSS at room temperature for 1 hour and then DTSSP (2 mM) (Thermo Fisher Scientific) was added to each the dishes to cross-link cell surface proteins for 30 mins and the reaction was stopped by Tris, pH 7.5 (20 mM). Plasma membrane fractionation was started 15 mins after the reaction was stopped and performed according to the instructions described in ref [37]. Briefly, after washing cells with ice-cold PBS twice, the subcellular fractionation buffer, which contains 250 mM sucrose, 20 mM HEPES, pH 7.4, 10 mM KCl, 1.5 mM MgCl_2_, 1 mM EDTA, and protease inhibitor (Sigma-Aldrich) and phosphatase inhibitor (Roche Life Science), was immediately added to cells to prepare cell lysates. The cell lysates were collected and centrifuged at 720 x g at 4°C for 10 mins followed by 10,000 x g at 4°C for 10 mins. The supernatant (mixture of cytosol and membrane fraction) was harvested and then subjected to ultracentrifugation at 100,000 x g at 6–7°C for 2 hours to generate pellet (membrane fraction). The supernatant was very carefully removed and the pellet was re-suspended with 400 μl to 1 ml of NP40 lysis buffer on ice for 30 mins. After centrifugation at 10,000 rpm at 4°C for 5 mins to remove un-dissolved debris, the solubilized membrane fraction was mixed with Protein A agarose to precipitate membrane-bound T-DM1 with cross-linked plasma membrane protein.

Plasma membrane protein extraction kit (101Bio) was employed to generate data presented in Figure 2E and Supplementary Figure 1C. According to the manufacturer, this kit separates total cellular components into four fractions: nuclei, cytosol, organelles and plasma membrane. The experiments were performed according to the manufacturer's protocol.

### Assays to determine binding of T-DM1 with CKAP5 independent of tubulin

#### Low pH treatment abolishes binding of T-DM1 to tubulin, but not CKAP5 (Figure 1F)

THLE2 or CHO-K1 cells (80–90% confluency in 10-cm dishes) were lysed by NP40-containing lysis buffer. After centrifugation to remove insoluble cellular debris, the whole cell lysates (WCL) were collected. The pH of WCL was adjusted to 3.0 with 1N HCl for 1–2 minutes and then quickly neutralized with 1N NaOH to 7.4. The WCL with or without low pH treatment was incubated with 100 μg T-DM1 for 1 hr followed by Protein A agarose precipitation. All procedures were performed either on ice or at 4°C.

#### Tubulin is not a component of T-DM1/CKAP5 cross-linked complex (Figure 1G)

4 × 10^6^ CHO-K1 cells were seeded in a 10-cm dish and cultured for 24 hrs. Subsequently, the cells from either one 10-cm plate or two 10-cm plates were subjected to cross-linking reaction described in the above section for Figure 2I and Supplementary Figure 1J. The cells were then washed with PBS, and the WCL was incubated with Protein A agarose beads for 2 hrs at 4°C. The Protein A agarose beads were washed with each NP40 lysis buffer and PBS three times and then incubated with IgG elution buffer at pH 3.0 (Thermo Fisher Scientific) followed by immediate neutralization to pH 7.5 using 1 M Tris, pH 11. The Protein A agarose beads were very carefully removed from the supernatant by a quick spin-down, and the samples of the supernatant were examined by Western blot analysis (Figure 1G, lane 3 from left, low pH treatment). Subsequently, fresh Protein A agarose beads was added to the supernatant again and incubated at room temperature for 30 mins to re-capture T-DM1/CKAP5 cross-linking complex. After washing Protein A agarose beads with PBS three times, 1 x Laemmli sample buffer was added to the Protein A agarose beads to elute all bound proteins followed by boiling at 95°C for 10 mins, and the samples were examined using Western blot analysis (Figure 1G, lane 4 from left, T-DM1 recapture).

### siRNA transfection

The siRNA transfection was performed using either Lipofecatmine 3000 (Thermo Fisher Scientific) or 4D-Nucleofector according to the manufacturer's procedures. Briefly, when Lipofectamine 3000 was used for transfection, 1.25 to 200 nM siRNA was transfected into 0.5 × 10^5^ THLE2 or CHO-K1 cells. When 4D-Nucleofector was used for the transfection, 250 to 500 nM siRNA was transfected into 1.0 × 10^6^ cells of THLE2 or CHO-K1 cells. 48 hrs post transfection, the cells were used for either immunostaining or Western blot analysis. The CKAP5 knocking-down efficiencies were evaluated by Western blot.

### Flow cytometry analysis

THLE2 or CHO-K1 cells were harvested by trypsinization and centrifugation. After washing with PBS, cells were fixed in 4% paraformaldehyde at room temperature for 20 mins and washed with TBS three times. Without permeabilization, cells were blocked with 10% donkey serum in TBS for 10 mins, and incubated with rabbit anti-CKAP5 antibody (cat# HPA040375, Sigma-Aldrich) and rabbit IgG as a control in the blocking buffer at room temperature for 1 hour. Cells were washed with TBS three times and incubated with anti-rabbit PE conjugated secondary antibody (Thermo Fisher Scientific) for 30 mins. Cells were washed again with PBS buffer for three times and re-suspended in 1 ml PBS, and analyzed on a BD FACSCallibur flow cytometer (BD Biosciences) according to the manufacturer's protocol. Data are presented in the form of histograms.

### Quantification and statistical analysis

GraphPad Prism was used for statistical studies. Statistical significance was determined by Student's *t*-test (^*^p-value < 0.05; ^**^p-value < 0.01; ^***^p-value < 0.001). Data are expressed as mean ± SD.

## SUPPLEMENTARY MATERIALS FIGURE AND VIDEOS












