# Supplementary material for: Payload of T-DM1 binds to cell surface cytoskeleton-associated protein 5 to mediate cytotoxicity of hepatocytes
Source: Oncotarget. 2018 Dec 14;9(98):37200–15. doi: 10.18632/oncotarget.26461 (PMC6324681; doi:10.18632/oncotarget.26461)
Supplement: Supplementary file 1 [file oncotarget-09-37200-s001.pdf]

Payload of T-DM1 binds to cell surface cytoskeleton-associated protein 5 to mediate cytotoxicity of hepatocytes

SUPPLEMENTARY MATERIALS

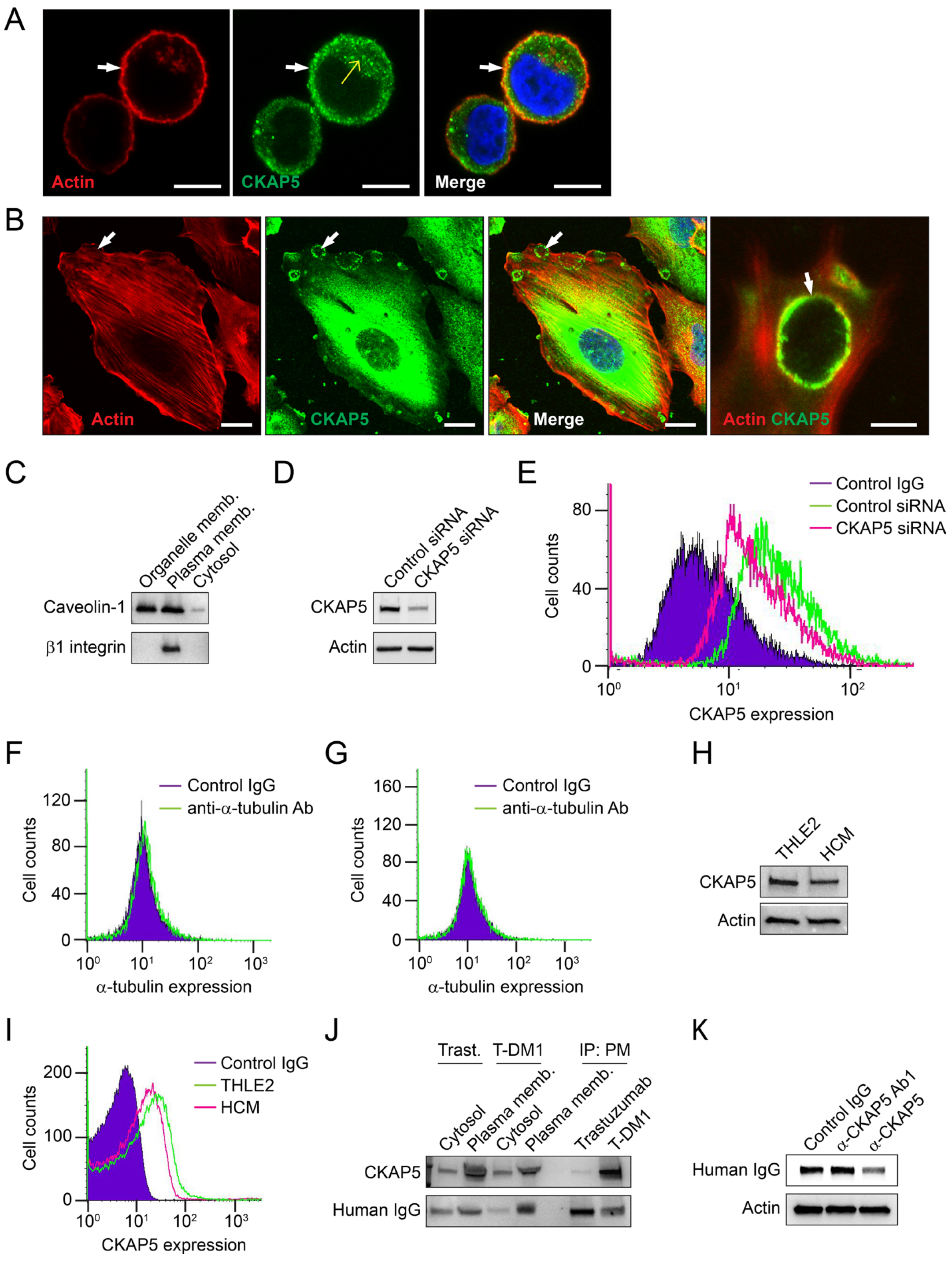

Supplementary Figure 1: CKAP5 is localized on the cell surface and concentrated on the membrane ruffles. (**A**) Fluorescent immunostaining of CKAP5 (green) and actin (red) at the cell edges in SKBR3 cells. Cells were permeabilized for the immunostaining. Scale bar, 10 µm. (**B**) Fluorescent immunostaining of CKAP5 (green) and actin (red) in THLE2 cells. Arrow indicates membrane ruffle. Cells were permeabilized for the immunostaining. Scale bar, 20 µm for three left panels and 5 µm for the first panel

from the left. (**C**) Subcellular fractionation of THLE2 cells shows that caveolin-1 is found in both organelle and plasma membrane, but β1 integrin is found only in plasma membrane. (**D**) Knocking-down efficiency of CKAP5 protein expression by either control or CKAP5 siRNA in CHO-K1 cells. (**E**) Flow cytometric histogram shows that CKAP5 protein expression is detected on the cell surface of CHO-K1 cells. The cells, which CKAP5 expression is silenced by siRNA, have a decreased signal intensity as compared with that of control cells. (**F** and **G**) Flow cytometric histogram shows that α-tubulin protein is not detected on the cell surface of THLE2 cells (**F**) or CHO-K1 cells (**G**). (**H**) Western blot shows that CKAP5 expression in THLE2 cells is higher than that in human cardiomyocytes (HCM). (**I**) Flow cytometry histogram shows that CKAP5 protein expression on the cell surface of THLE2 cells is higher than that in HCM cells. (**J**) T-DM1 binds to CKAP5 from the plasma membrane fraction of CHO-K1 cells. After incubating 250 µg/ml trastuzumab and T-DM1 with CHO-K1 cells in HBSS at RT for 1 hour, cell surface proteins were cross-linked by DTSSP, and the WCLs were subjected to subcellular fractionation into cytosol and plasma membrane (PM) fraction. PM fraction was solubilized and then subjected to immunoprecipitation. (**K**) Anti-CKAP5 antibody blocks the binding of T-DM1 to the THLE2 cells. Amounts of cell-bound T-DM1 are examined in WCL of THLE2 cells which were pre-incubated with 10 µg/ml rabbit IgG, anti-CKAP5 Ab1 (cat# HPA039377, Sigma-Aldrich), or anti-CKAP5 (cat# HPA040375, Sigma-Aldrich) at 37°C for 1 hour, and cells then were incubated with 100 µg/ml T-DM1 at 37°C for 1 hour. The amount of cell-bound T-DM1 was reduced in the cells pre-treated with anti-CKAP5, compared to that in the cells pre-treated with control IgG or anti-CKAP5 Ab1 antibody.

Supplementary Video 1: Human hepatocytes (THLE2) cells were incubated at 37°C for 45 mins, and then rhodamine-conjugated wheat germ agglutinin (WGA) was added to cell culture for 15 mins. After washing cells with the cell culture media twice, time-lapse recording was performed for 30 mins using a Leica SP8 DMI 6000. Images shown were taken at 0, 15, and 30 min time-points. See Supplementary_Video_1

Supplementary Video 2: Human hepatocytes (THLE2) cells were incubated with 100 µg/ml trastuzumab at 37°C for 45 mins, and then rhodamine-conjugated wheat germ agglutinin (WGA) was added to cell culture for 15 mins. After washing cells with the cell culture media twice, time-lapse recording was performed for 30 mins using a Leica SP8 DMI 6000. Images shown were taken at 0, 15, and 30 min time-points. See Supplementary_Video_2

Supplementary Video 3: Human hepatocytes (THLE2) cells were incubated with 100 µg/ml brentuximab vedotin at 37°C for 45 mins, and then rhodamine-conjugated wheat germ agglutinin (WGA) was added to cell culture for 15 mins. After washing cells with the cell culture media twice, time-lapse recording was performed for 30 mins using a Leica SP8 DMI 6000. Images shown were taken at 0, 15, and 30 min time-points. See Supplementary_Video_3

Supplementary Video 4: Human hepatocytes (THLE2) cells were incubated with 100 µg/ml T-DM1 at 37°C for 45 mins, and then rhodamine-conjugated wheat germ agglutinin (WGA) was added to cell culture for 15 mins. After washing cells with the cell culture media twice, time-lapse recording was performed for 30 mins using a Leica SP8 DMI 6000. Images shown were taken at 0, 15, and 30 min time-points. See Supplementary_Video_4

Supplementary Video 5: Human hepatocytes (THLE2) cells were incubated with 100 µg/ml α-CD22-DM1 at 37°C for 45 mins, and then rhodamine-conjugated wheat germ agglutinin (WGA) was added to cell culture for 15 mins. After washing cells with the cell culture media twice, time-lapse recording was performed for 30 mins using a Leica SP8 DMI 6000. Images shown were taken at 0, 15, and 30 min time-points. See Supplementary_Video_5
